# Effects of exercise-cognitive dual-task training on cognitive frailty in older adults: a randomized controlled trial

**DOI:** 10.3389/fnagi.2025.1639245

**Published:** 2025-09-16

**Authors:** Xiaoxing Lai, Hongwei Zhu, Yonghua Cai, Baoyu Chen, Yang Li, Hongdi Du, Liping Zhang, Wenwen Wang, Shuxian Li, Xiaopeng Huo

**Affiliations:** ^1^Department of Neurology, Peking Union Medical College Hospital, Beijing, China; ^2^Nursing Department, Peking Union Medical College Hospital, Beijing, China; ^3^Peking University Sixth Hospital, Peking University Institute of Mental Health, NHC Key Laboratory of Mental Health (Peking University), Beijing, China; ^4^Department of Health Care, Peking Union Medical College Hospital, Beijing, China

**Keywords:** cognitive frailty, older adults, dual-task training, exercise, cognition

## Abstract

**Objective:**

This study aims to investigate the effects of exercise-cognitive dual-task training on frailty status, cognitive function, physical performance, and dual-task cognitive load in older adults with Cognitive frailty (CF) over a 16-week intervention period.

**Methods:**

This randomized controlled trial enrolled older adults with CF at community health service center in Chaoyang District, Beijing, between February and March 2024. Participants were randomly assigned to either the dual-task training group or the health education group in a 1:1 ratio. The dual-task training group received an exercise-cognitive dual-task training program, while the health education group received information on CF, including its symptoms, risk factors, and non-pharmacological prevention and treatment strategies. The primary outcomes were frailty status, while the secondary outcomes included cognitive function, balance and gait function, walking ability, and dual-task cognitive load.

**Results:**

A total of 72 participants (35 males) were enrolled, including 36 individuals (mean age: 74.81 ± 8.23 years, 17 males, mean BMI: 21.38 ± 2.83 kg/m^2^) in the dual-task training group, and 36 individuals (mean age: 76.50 ± 7.75 years, 18 males, mean BMI: 22.18 ± 2.12 kg/m^2^) in the health education group. Participants (n = 72) were 75.66 ± 7.9 years old; 48.6% (35/72) were male and 51.4% (37/72) were female. Following the intervention, the dual-task training group exhibited significant improvements compared to the health education group in the Tilburg Frailty Index (5.14 ± 0.99 vs. 7.36 ± 1.07, *p* < 0.001) and Montreal Cognitive Assessment scores (27.25 ± 2.41 vs. 23.47 ± 1.87, *p* < 0.001). Additionally, the dual-task training group demonstrated superior outcomes in the Performance-Oriented Mobility Assessment (POMA) scores (24.64 ± 5.50 vs. 17.39 ± 4.38, *p* < 0.001), Time Up and Go Test (TUGT) indicators (10.66 ± 1.76 vs. 12.01 ± 2.21, *p* < 0.05), and cognitive load measures (all *p* < 0.05).

**Conclusion:**

Exercise-cognitive dual-task training may effectively improve frailty status, cognitive function, physical performance, and dual-task cognitive load in older adults with CF, suggesting its potential for broader application in this population.

**Clinical trial registration:**

http://www.chictr.org.cn/, ChiCTR2400080105.

## Introduction

Cognitive frailty (CF) is a heterogeneous geriatric syndrome primarily characterized by the coexistence of physical frailty and cognitive impairment, excluding dementia. Studies indicate that the prevalence of CF ranges from 4.4 to 39.7% (Facal et al., 2019), significantly affecting the quality of life and social participation of older adults while imposing substantial economic and social burdens on public health systems (Qiu et al., 2022). A cohort study involving 2,375 individuals in Singapore revealed that, compared to older adults without CF, those with CF exhibited a 12-fold increase in disability rates, a 5-fold increase in the incidence of reduced quality of life, and a 5-fold higher risk of mortality (Feng et al., 2017). Nevertheless, older adults with CF may regain normal physical and cognitive functions through appropriate intervention (Vatanabe et al., 2022). Early screening, accurate assessment, and timely intervention are critical to prevent adverse health outcomes such as dementia and falls. Dual-task training has emerged as a promising approach, offering potential benefits for improving both cognitive and physical functions in older adults with CF.

Dual tasking refers to the simultaneous execution of two tasks with distinct goals that can be performed and measured independently (McIsaac et al., 2015). Studies suggest that the ability to manage tasks requiring both attentional control and motor-cognitive functions declines with age, particularly in individuals with neurodegenerative diseases such as Parkinson’s disease and Alzheimer’s disease (Alexander et al., 2012). Exercise-cognitive dual-task training focuses on the interaction between cognitive and motor control, simulating multitasking scenarios encountered in daily life. By promoting neurogenesis and neuronal proliferation, it improved both cognitive performance and physical function (Trombini-Souza et al., 2020). In addition, dual-task training enhanced both cognitive and motor functions through a series of biological and neural mechanisms (Anderson-Hanley et al., 2018), including changes in brain metabolism (oxygen and glucose) and neurochemical activity (dopamine and neurotrophins).

Existing research on dual-task training has focused mainly on improving gait and balance in healthy older adults, stroke survivors, and individuals with Alzheimer’s disease (Zhang et al., 2019; Tasseel-Ponche et al., 2023; de Barros et al., 2021). Fewer studies have examined cognitive outcomes, but these have reported encouraging results. Even so, the overall cognitive efficacy of dual-task training in older adults remains unsettled: a recent randomized controlled trial found that dual-task training yields cognitive benefits similar to those achieved by sequential exercise or cognitive training alone (Gavelin et al., 2021). At the same time, questions persist about the safety and tolerability of dual-task training in older adults. Rezola-Pardo et al. (2022) observed that adults over 70 assigned to dual-task training; experienced more falls than peers performing multicomponent exercise only, underscoring potential risks. Further research involving a diverse range of participants is necessary to comprehensively assess the impact of dual-task training on older adults with CF.

Although dual-task training shows promise for mitigating CF, empirical data in older adults with CF remain scarce. Moreover, existing protocols predominantly target physical outcomes, leaving the cognitive component of CF largely unaddressed. Few studies have examined the application of dual-task training in older adults with CF. Given the reversible nature of CF, this study developed an exercise-cognitive dual-task training program tailored for older adults with CF, emphasizing the interplay between physical and cognitive functions and observing dual-task cognitive load. This study was conducted among older adults in community settings in China, providing new strategies for exercise-cognitive rehabilitation in this population.

In a single-blind, parallel-group RCT we compared a 16-week dual-task exercise program (each time for 30–40 min, twice a week) with usual-care control in community-dwelling Chinese older adults with cognitive frailty. The outcomes of this study were frailty status, cognitive function, physical performance, and dual-task cognitive load. This study was highly likely to be the first RCT to test dual-task training specifically in Chinese older adults with cognitive frailty. Furthermore, the dual-task intervention designed in this study integrates specific cognitive and physical training to simulate the multitasking scenarios commonly encountered in daily life. It was hypothesized that the dual-task training could improve frailty status, cognitive function, physical performance, and dual-task cognitive load in older adults with CF compared with health education.

## Methods

### Study design

Single-blind randomized controlled trail (Trial registration: ChiCTR, ChiCTR2400080105). This study was enrolled at a community health service center Chaoyang District, Beijing, China, between 1 February and 31 March 2024, followed by a 16-week intervention. The baseline assessments were completed before the start of the 16-week intervention on April 2024.

### Participants

The study was conducted and reported according to the CONSORT (Consolidated Standards of Reporting Trials) guidelines. The participants were recruited through health education and propaganda posters on the bulletin board of the pension agency. Each eligible participant was fully informed of the study’s aims, processes and potential risks. This study was approved by the Ethics Committee of the Peking Union Medical College Hospital (No. ZS-2943), and all participants signed the informed consent form.

Inclusion Criteria: (1) age ≥ 60 years; (2) meet the criteria for CF: subjective cognitive decline (SCD) without a formal diagnosis of dementia, with a Clinical Dementia Rating (CDR) score of 0.5, a Montreal Cognitive Assessment (MoCA) score below 26 (with an additional point for ≤12 years of education), and a Frailty Phenotype Scale score of ≥3; (3) conscious and able to answer questions independently; (4) provision of informed consent and voluntary participation.

Exclusion Criteria: (1) severe aphasia or sensory impairments (e.g., visual or auditory dysfunction); (2) severe organ dysfunction, malignancies, or terminal illness; (3) acute exacerbation of chronic cardiovascular, cerebrovascular, or pulmonary diseases; (4) neuro-musculoskeletal disorders causing limb mobility impairment; (5) severe trauma or other contraindications for physical activity; (6) inability to read, write, or cooperate with testing.

Dropout Criteria: Participants who were enrolled but did not complete the full intervention were considered dropouts under the following conditions: voluntary withdrawal, poor compliance (less than 80% participation in required sessions), or deterioration in physical condition preventing continued participation. Reasons for participant withdrawal and the time of withdrawal were documented.

### Sample size

Sample size calculation was based on the “two-sample mean comparison” method.


$$n=\frac{\;{\left(Z_{\alpha }+Z_{\beta }\right)}^{2}\times 2\sigma^{2}\;}{\delta^{2}}$$

The sample size was calculated using the following parameters: *α* = 0.05, *β* = 0.10, *μ*_α_ = 1.96, and *μ*_β_ = 1.282, employing a two-sided test. The frailty score was selected as the primary outcome measure of this study. Based on preliminary, unpublished experiments conducted by the research team, the mean difference (*δ*) in frailty scores was determined to be 1.93, with a standard deviation (*σ*) of 2.31. Consequently, the sample size required for each group was calculated to be 30 participants. Accounting for a 10% dropout rate, the final sample size for each group was determined to be 36 participants per group, resulting in a total of 72 participants.

### Randomization

In this study, a random number table was used to generate non-repeating random numbers. Participants assigned even numbers were allocated to the health education group, while those assigned odd numbers were allocated to the dual-task training group, with each group consisting of 36 participants. If adjustments were required to balance the number of participants (“*n*”), additional random numbers were drawn until an equal distribution was achieved. The final random number sequence determined the allocation of participants to their respective groups.

### Interventions

Based on a comprehensive literature review, this study developed an exercise-cognitive dual-task training intervention program tailored for older adults with CF. The program was adapted from the “NCGG Home Exercise Program for Older People (NCGG-HEPOP)” published by the National Center for Geriatrics and Gerontology in Japan (Osawa et al., 2020), to better suit the health conditions of older adults with CF (Supplementary Table 1).

Duration and frequency: 16 consecutive weeks, three sessions per week (suggest on Mon/Wed/Fri), each session 50–60 min. Exercise intensity was determined by heart rate, with the appropriate range calculated as (220-age) × (60–80%), ensuring participants did not experience significant fatigue.

Session structure: (1) Warm-up (5–10 min): Seated marching, shoulder rolls, dynamic stretching, and 1-min single-task walking at self-selected pace. (2) Exercise-Cognitive Dual-Task Training Intervention Protocol (40 min): 10 stations, 2 min each, 2 min transition. (3) Cool-down (5–10 min): Static stretching, diaphragmatic breathing, and 30-s single-task walk re-test for safety check.

Training Supervision: During the first 2 weeks, group interventions were conducted at the community health center, where participants received face-to-face guidance on dual-task training. The study were led by two medical staff who completed a 24-h, certified and standard course to ensured consistent delivery. The course included protocol walk-throughs, scripted cueing, safety drills, and three observed mock sessions. Simultaneously, participants’ family members or companions also received education to ensure the correct continuation of dual-task training at home. The instructional videos, demonstrated by rehabilitation therapists, were produced and converted into QR codes. A “Dual-Task Training Instruction Manual for older adults with CF” containing QR codes linking to these instructional videos was provided to participants. By scanning the QR codes using the WeChat application (Tencent Holdings Limited), a widely used social media platform in China, participants could access the videos and follow the dual-task training instructions.

From the third to the sixteenth week, home-based interventions were conducted with the assistance of family members and feedback was provided through WeChat. Adherence was defined as the completion of at least 80% of the prescribed sessions (i.e., ≥39 out of 48 sessions over 16 weeks). Weekly check-ins via the WeChat Mini Program were logged and reviewed to monitor participant compliance. To ensure adherence to the intervention, the research team also performed four home visits (in the 4th, 8th, 12th, and 16th weeks) and utilized the group clock-in function on the WeChat application. With family assistance, participants completed a weekly clock-in via WeChat, which allowed the research team to monitor adherence by accessing real-time clock-in records through the WeChat Mini Program, including participant numbers and clock-in times. Measures were taken to ensure participants maintained proper and standardized training at home.

Exercise monitoring and safety: The training program prioritized participant safety by incorporating gradual progression, the use of supportive tools (e.g., stable tables or handrails), and ensuring constant accompaniment during exercises. In this study, dual-task training was conducted at a moderate-to-low intensity level. Participants were instructed to monitor their exercise intensity using a validated chest-strap heart-rate monitor (Polar H10) provided during group interventions at the community health center. For home sessions, participants or caregivers measured radial pulse manually before and after sessions. In addition, the Borg Rating of Perceived Exertion (RPE) scale was used to ensure moderate intensity (RPE 12–14). Exercise was paused if heart rate exceeded 85% of age-predicted maximum for >30 s or if RPE exceeded 16. Participants were also instructed to stop training immediately if they experienced any discomfort, such as dizziness, shortness of breath, or chest pain. Additionally, participants and their families were provided with contact information for the community health center staff for home visits, vital sign monitoring, and prompt management of any potential health issues.

The health education group received health education focusing on CF, including clinical manifestations, associated risk factors, and non-pharmacological prevention and treatment strategies. Following the intervention period, participants in the health education group were offered the same dual-task training program as the dual-task training group, based on personal preferences.

### Outcome measurements

The primary outcome was frailty status, which was assessed by the Tilburg Frailty Indicator (TFI) (Gobbens et al., 2010). This scale evaluates three dimensions: physical frailty, psychological frailty, and social frailty, comprising a total of 15 items. The total score ranges from 0 to 15, with a score of 5 or higher indicating frailty. Higher scores reflect greater frailty. The Cronbach’s αcoefficient of the Chinese version of TFI is 0.75, and the test–retest reliability is 0.76, indicating good psychometric properties in terms of reliability and validity when applied in China (Si et al., 2018).

The secondary outcomes included assessments of cognitive function, balance and gait function, walking ability, and dual-task cognitive load. Cognitive function was evaluated using Montreal Cognitive Assessment (MoCA) (Nasreddine et al., 2005), which assesses multiple cognitive domains including visuospatial abilities, naming, memory, attention, language fluency, abstraction, delayed recall, and orientation. The total score ranges from 0 to 30, with higher scores indicating better cognitive function. The MoCA-Beijing version has demonstrated excellent internal consistency (Cronbach’s *α* = 0.848) and good test–retest reliability (ICC = 0.959) among Chinese older adults (Chen et al., 2015).

Balance and gait function were measured using the Performance-Oriented Mobility Assessment (POMA) (Yelnik and Bonan, 2008), which consists of two subscales: balance testing (9 items, 16 points) and gait testing (8 items, 12 points), with a maximum score of 28 points. Lower total scores indicate poorer motor function. The Cronbach’s αcoefficient of the Chinese version of POMA scale is 0.887, and the test–retest reliability is 0.886 (Gao et al., 2014). Walking ability was assessed using the Time Up and Go Test (TUGT), which measures the time (in seconds) required for a participant to rise from a standard armchair, walk 3 m, return to the chair, and sit down. The test was performed three times, and the average time was recorded. The Cronbach’s α coefficient of the Chinese version of TUGT is 0.980, and the test–retest reliability is 0.934 (Yi et al., 2022; Bao et al., 2021).

The dual-task cognitive load was assessed using a writing and cognitive task performed with a Bluetooth smart pen (Moleskine Pen+Ellipse) and Ncoded paper technology. This smart pen, equipped with a miniature camera at the tip, simultaneously records both writing and drawing content while also capturing audio. Each stroke is recorded in real-time and transmitted to an electronic device via the Moleskine Notes application. The Moleskine Pen+Ellipse smart pen has been previously validated for handwriting kinematics and cognitive task timing in aging populations (Ma et al., 2020). Prior to the study, participants underwent a familiarization session to reduce potential learning effects and ensure usability. The dual-task cognitive load assessment consisted of two sequential tasks (Vandenbossche et al., 2014). (1) Participants wrote the sentence “Xiao Guo wants to exchange his red flower for Xiao Ge’s yellow flower” at their normal writing speed. (2) Participants completed a sound recognition cognitive task requiring attentional focus by identifying the number of tapping sounds delivered at 3-s intervals. Participants then performed both tasks simultaneously—writing while completing the sound recognition task—without prioritizing either task. To minimize memory interference, a different sentence, “Xiao Ge wants to exchange his yellow flower for Xiao Guo’s red flower.” was used during the dual-task condition. The researcher recorded the time required for single-task writing, the time for dual-task writing, the number of correct responses during the single listening task, and the number of correct responses during the dual-task condition. Dual-task cost (DTC) was calculated to quantify cognitive-motor interference using the formula: DTC = (single-task performance – dual-task performance)/single-task performance. Higher absolute DTC value indicate greater cognitive-motor interference, reflecting poorer performance in individual tasks, while lower DTC values suggest better performance (Leone et al., 2015).

### Statistical analysis

Data entry was performed using EpiData 3.1 software (EpiData Association, Odense, Denmark), while statistical analysis was conducted using SPSS 22.0 software (IBM Corp., Armonk, NY, United States). All data were analyzed using the intention-to-treat principle to minimize bias in outcome assessments. Data normality were tested before selecting the statistical methods. Quantitative data were expressed as mean ± standard deviation (SD), and comparisons were performed using the t-test. Categorical data were expressed as *n* (%), and comparisons were made using the *χ^2^* test or Fisher’s exact probability method where appropriate. Statistical significance was set at two-sided *p* < 0.05.

## Results

### Baseline characteristics

**Figure 1:**
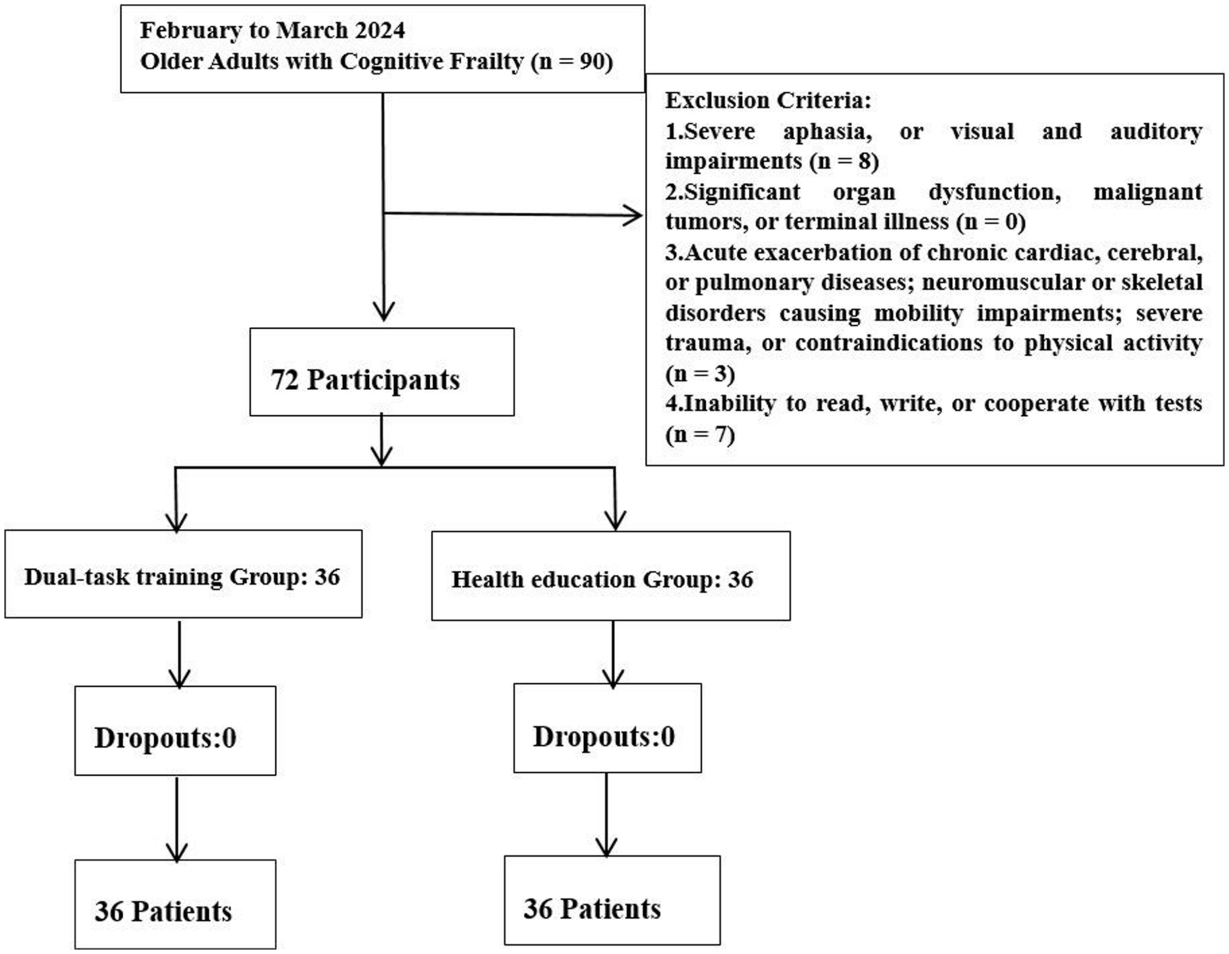
Flowchart of the screening research population.

A total of 72 participants (35 males) were included in the study (Figure 1), with 36 participants in each group (dual-task training group and health education group). No participants withdrew from the study. The mean age of participants in dual-task training group was 74.81 ± 8.23 years, while mean age of health education group was 76.50 ± 7.75 years. In the Dual-task training Group (*n* = 36), there were 17 males (47.22%) and 19 females (52.78%), the mean score of Activities of Daily Living (ADL) was 72.36 ± 12.32, and the mean Body Mass Index (BMI) was 21.38 ± 2.83 kg/m^2^. In the health education group (*n* = 36), there were 18 males (50.00%) and 18 females (50.00%), the mean score of ADL was 71.92 ± 10.91, and the mean BMI was 22.18 ± 2.12 kg/m^2^. In the dual-task training group, 20 out of 36 participants (56%) had completed middle or high school education, compared to 19 out of 36 participants (53%) in the health education group. No significant differences were observed between the two groups in terms of gender (*p* = 0.814), age (*p* = 0.371), education level (*p* = 0.779), daily living activities (*p* = 0.872), body mass index (BMI) (*p* = 0.182), or chronic diseases (*p* = 0.385) (Table 1).

**Table 1:** Comparison of general information between the two groups.

Variables	Dual-task training group (*n* = 36)	Health education group (*n* = 36)	*P*
Gender, *n* (%)			0.814
Male	17(47.22)	18(50.00)	
Female	19(52.78)	18(50.00)	
Age (years)	74.81 ± 8.23	76.50 ± 7.75	0.371
Educational, *n* (%)			0.779
Primary school or below	14(38.89)	16(44.44)	
Middle school and high school	20(55.56)	19(52.78)	
College and above	2(5.55)	1(2.78)	
Activities of daily living	72.36 ± 12.32	71.92 ± 10.91	0.872
Body mass index (kg/m^2^)	21.38 ± 2.83	22.18 ± 2.12	0.182
Number of chronic diseases	4.17 ± 1.03	4.39 ± 1.13	0.385

### Primary outcome

No participants withdrew from either group during the intervention. Prior to the intervention, no significant differences in TFI scores was observed between the dual-task training group and the health education group (7.22 ± 0.9 vs. 7.06 ± 1.09, *p* = 0.482). After the intervention, the TFI frailty score in the dual-task training group significantly decreased (7.06 ± 1.09 vs. 5.14 ± 0.99, *p* < 0.001). A statistically significant difference was also observed between the dual-task training group and the health education group post-intervention (5.14 ± 0.99 vs. 7.36 ± 1.07, *p* < 0.001). In contrast, no significant difference was observed in the health education group between pre- and post-intervention assessments (*p* > 0.05) (Table 2).

**Table 2:** Comparison of frailty and cognitive function indicators between the two groups before and after intervention.

Stage	Variables	Dual-task training group (*n* = 36)	Health education group (*n* = 36)	*P*	Cohen’s *d*
Prior to the intervention	TFI scores	7.06 ± 1.09^**^	7.22 ± 0.9	0.482	−0.16
	MoCA scores	23.22 ± 1.62^**^	23.58 ± 1.83	0.378	−0.06
Post-Intervention	TFI scores	5.14 ± 0.99	7.36 ± 1.07	<0.001	−2.11
	MoCA scores	27.25 ± 2.41	23.47 ± 1.87	<0.001	1.29

**P < 0.001.

### Secondary outcome

Prior to the intervention, no statistically significant differences were observed between the two groups across all secondary outcome measures (*p* > 0.05). Following the intervention, the MoCA score in the dual-task training group significantly increased (23.22 ± 1.62 vs. 27.25 ± 2.41, *p* < 0.001). Significant improvements were also observed in the POMA score (24.64 ± 5.50 vs. 17.94 ± 4.60, *p* < 0.001), and in the TUGT results (10.66 ± 1.76 vs. 12.49 ± 1.66, *p* < 0.001). Comparisons between the dual-task training group and the health education group post-intervention also showed statistically significant differences in MoCA score (27.25 ± 2.41 vs. 23.47 ± 1.87, *p* < 0.001), POMA score (24.64 ± 5.50 vs. 17.39 ± 4.38, *p* < 0.001) and TUGT score (10.66 ± 1.76 vs. 12.01 ± 2.21, *p* < 0.05). In the health education group, no significant changes were found between pre- and post-intervention comparisons (*p* > 0.05) (Table 3).

**Table 3:** Comparison of physical function and dual-task indicators between the two groups before and after intervention.

Stage	Variables	Dual-task training group (*n* = 36)	Health education group (*n* = 36)	*P*
Prior to the intervention	POMA scores	17.94 ± 4.60^**^	17.47 ± 4.48	0.660
	TUGT(s)	12.49 ± 1.66^**^	11.78 ± 2.08	0.114
	Single task writing time (s)	42.69 ± 5.32^**^	40.58 ± 6.91	0.151
	Dual task writing time(s)	50.94 ± 8.78^**^	49.44 ± 8.17	0.456
	Single listening accuracy	14.83 ± 2.21^**^	15.19 ± 2.48	0.517
	Writing and listening accuracy	12.56 ± 2.22^**^	12.64 ± 2.17	0.872
	Writing time DTC	0.44 ± 0.12^**^	0.45 ± 0.1^n^	0.613
	Writing and listening DTC	0.06 ± 0.02^**^	0.07 ± 0.02	0.080
Post-intervention	POMA scores	24.64 ± 5.50	17.39 ± 4.38	<0.001
	TUGT(s)	10.66 ± 1.76	12.01 ± 2.21	0.005
	Single task writing time (s)	39.03 ± 5.47	41.89 ± 7.13	0.044
	Dual task writing time (s)	42.53 ± 9.03	48.97 ± 8.28	0.001
	Single listening accuracy	16.83 ± 2.13	14.31 ± 2.58	0.003
	Writing and listening accuracy	15.17 ± 2.09	12.89 ± 2.17	<0.001
	Writing time DTC	0.32 ± 0.12	0.44 ± 0.14	<0.001
	Writing and listening DTC	0.02 ± 0.01	0.05 ± 0.01	<0.001

**P < 0.001, n, represents the number of participants included in the specific analysis for each variable.

Significant differences were also noted between the two groups in dual-task performance post-intervention, including single-task time (39.03 ± 5.47 vs. 41.89 ± 7.13, *p* < 0.05), dual-task time (42.53 ± 9.03 vs. 48.97 ± 8.28, *p* < 0.05), single listening accuracy (16.83 ± 2.13 vs. 14.31 ± 2.58, *p* < 0.05), number writing and listening accuracy (15.17 ± 2.09 vs. 12.89 ± 2.17, *p* < 0.001), writing time DTC (0.32 ± 0.12 vs. 0.44 ± 0.14, *p* < 0.001), and writing and listening time DTC (0.02 ± 0.01 vs. 0.05 ± 0.01, *p* < 0.001). In the dual-task training group, significant improvements in dual-task indicators were observed before and after the intervention (p < 0.05). While no significant differences were found in the health education group before and after the intervention (*p* > 0.05) (Table 3).

## Discussion

The study aimed to investigate the effects of exercise-cognitive dual-task training in older adults with CF. Our study revealed several important findings. This study demonstrated that the dual-task training group significantly improved TFI scores compared to the health education group. Additionally, MoCA scores, TUGT results, POMA scores, and cognitive load measures were significantly improved. These findings suggest that exercise-cognitive dual-task training is effective in enhancing frailty status, cognitive function, physical function, and dual-task cognitive performance in older adults with cognitive frailty.

This study reinforces that exercise-cognitive dual-task training is a potent and reproducible strategy for mitigating frailty in older adults, aligning with and extending prior evidence. A recent network meta-analysis involving 1,110 patients demonstrated that dual-task training is beneficial in managing frailty (Zhang et al., 2023). Frailty and cognitive impairment are interrelated, contributing to the development and progression of frailty. Evidence indicates that frailty is a dynamic condition, and targeted interventions for cognitively frail individuals may delay or even reverse its progression (Panza et al., 2018). Multimodal interventions have also been shown to alleviate frailty and reduce adverse health outcomes (Chen et al., 2020). The exercise component of dual-task training integrates aerobic, balance, and flexibility exercises, while cognitive training enhances short-term memory, attention, and problem-solving abilities. Furthermore, dual-task training also promotes skeletal muscle growth by stimulating muscle protein synthesis and reducing catabolism, thereby improving muscle function in older adults. By increasing muscle mass and strength, dual-task training enhances frailty status (Aguirre and Villareal, 2015). Thus, for cognitively frail older adults, this dual-task approach enhances both cognitive function and physical ability.

After age of 65, brain volume decreases by approximately 0.5 to 1% annually, while hippocampal volume declines by 1–2% per year, increasing the risk of cognitive impairment (Pascoal et al., 2020). This study demonstrated that improved MoCA scores reflect indicated enhanced cognitive function in cognitively frail older adults following exercise-cognitive dual-task training, consistent with findings by Bae et al. (2019). Similarly, a recent Chinese study also reported that the exercise group showed improved MoCA scores after 3 or 6 months of intervention (Ye et al., 2020). This cognitive improvement may be attributed to motor relearning through repetitive and varied training, which facilitates the formation of new synaptic chains and neural circuits, thus enhancing central nervous system function. Additionally, skeletal muscle acts as an important endocrine organ, with exercise stimulating the secretion of cathepsin B from muscles, which crosses the blood–brain barrier and increases hippocampal brain-derived neurotrophic factor levels. This process regulates synaptic plasticity and neuronal connectivity, thereby improving memory (De la Rosa et al., 2019). Long-term dual-task training enables older adults to better allocate cognitive resources, enhancing time management and attention-shifting strategies. This leads to improved cortical activation in regions responsible for executive functions, and significantly enhancing both attention and executive function. Although the present trial was not designed for mechanistic dissection, the current study suggests that simultaneous motor and cognitive challenges up-regulate brain-derived neurotrophic factor (BDNF), and increase functional connectivity within the prefrontal–hippocampal network. These neuroplastic changes are thought to underlie the dual gains in cognitive function, physical performance we observed.

Older adults with CF often reach the limits of their ability to allocate attentional resources, which reduces attention to postural control and increases the risk of falls (Ko et al., 2018). A systematic review of 11 trials involving 322 participants found that dual-task training significantly improved gait speed, cadence, motor symptoms, and balance (Li et al., 2020). Therefore, in this study, exercise-cognitive dual-task training was implemented in older adults with CF to enhance their gait and balance function. The intervention group performed cognitive tasks, such as numerical addition and semantic naming, while engaging in seated or standing exercises. The dual-task training optimized cognitive allocation strategies, improved cognitive switching speed, increased the volume of the prefrontal cortex, delayed the atrophy of brain regions such as the hippocampus, and enhanced memory and executive functions (Northey et al., 2018). By simulating real-life multitasking scenarios, dual-task training strengthens the interaction between cognition and motor control, improving the ability to perform multiple tasks simultaneously and reducing the risk of falls.

Cognitive load refers to the amount of mental resources required to perform a task, defined as the relationship between task demands and available cognitive capacity. The level of cognitive load directly influences task performance (Young et al., 2015). Writing, a uniquely human behavior, is a high-skill, rhythmic fine motor activity. Successful writing requires the coordination of cognitive, sensory, and motor systems while utilizing the brain’s cognitive and memory resources (Bisio et al., 2017).

In this study, the dual-task writing test indicated that the dual-task training group significantly reduced dual-task writing time and increased the number of correct auditory recognition tasks compared to the health education group. This improvement may be attributed to the beneficial effects of an appropriate cognitive load on work efficiency, consistent with the findings of Zhang et al. (2020). During ambulation, adding a secondary task may enhance postural stability, as increased cognitive load could improve postural control.

After the intervention, the dual-task training group demonstrated significantly shorter dual-task writing time, lower dual-task writing DTC values, and a greater number of correct auditory recognition responses compared to the health education group. When measuring task interference using DTC, it was observed that the cognitive load on writing was more pronounced in the dual-task training group, while the impact of listening on cognitive load was less substantial. During dual-task performance, individuals typically prioritize more complex tasks while allocating fewer resources to simpler ones, with writing being a more common task in daily life (Pashler, 1994). Previous studies suggest that while task prioritization varies among individuals, it can be influenced by external instructions (Jansen et al., 2016). It’s possible that the researchers emphasized the complexity of the listening task, leading participants to perceive it as a priority.

This study demonstrated that dual-task training significantly improves physical performance and cognitive function in the older adults, underscoring its potential application in community health management. Our findings underscore the importance of additional research in this area. Future research should focus on its long-term effects of dual-task training on CF and dementia prevention through larger, more diverse samples and comprehensive medical assessments.

### Strength and limitations

This study possesses several strengths. Our study is among the first randomized controlled trials to evaluate dual-task training specifically in Chinese community-dwelling older adults with confirmed cognitive frailty, thereby addressing an under-represented and high-risk population that existing literature has largely overlooked. Furthermore, the dual-task intervention designed in this study integrates specific cognitive and physical training to simulate the multitasking scenarios commonly encountered in daily life. This intervention protocol is highly practical, efficient, safe, and widely replicable, making it suitable for implementation in community settings as well as in nursing homes and care facilities.

However, this study also has several limitations. First, due to time and resource constraints, the sample was drawn from a single source, consisting of older adults from communities in Beijing, which may limit the representativeness of our sample and restrict external validity. Additionally, the relatively small sample size may limit the generalizability of the findings. Future prospective studies involving diverse populations across various regions and socioeconomic backgrounds are necessary to evaluate the sustained effects of dual-task interventions on improving CF. Secondly, although our 16-week intervention produced significant improvements in cognitive frailty, we did not collect follow-up data beyond the post-intervention assessment. We are currently designing a 12-month longitudinal extension that will track cognitive and functional outcomes to clarify the durability and preventive potential of dual-task training in this population. Finally, cognitive outcomes were assessed with brief cognitive screening instrument, but were not corroborated by objective neuroimaging or biomarkers. Because only a cognitive screening measure was employed, more comprehensive neuropsychological evaluations are required before definitive conclusions can be drawn about the cognitive effects of the dual-task training. Future multicenter trials are needed to validate our findings and enhance generalizability. Specifically, the studies should integrate objective medical assessments, such as functional brain imaging and biomarker analysis, to quantify intervention-related changes in neuroplasticity and physical activity.

## Conclusion

Overall, the results suggest that exercise-cognitive dual-task training effectively improves frailty, cognitive function, physical mobility, and cognitive load in older adults with CF by optimizing cognitive allocation, enhancing switching speed, and increasing coordination between motor and cognitive tasks. These findings might be used as a valuable reference for specific recommendations on cognitive and physical training, safety and effective exercise plans, thus helping to promote healthy aging in the older population. Long-term, multi-center prospective studies are needed to confirm and extend our findings. Such studies would provide stronger evidence supporting the inclusion of dual-task training interventions in older adults CF rehabilitation programs, also offer further insights into the underlying mechanisms driving the benefits of dual-task training.

## Data Availability

The original contributions presented in the study are included in the article/Supplementary material, further inquiries can be directed to the corresponding authors.
