# Supplementary material for: Effects of exercise-cognitive dual-task training on cognitive frailty in older adults: a randomized controlled trial
Source: Front Aging Neurosci. 2025 Sep 16;17:1639245. doi: 10.3389/fnagi.2025.1639245 (PMC12479488; doi:10.3389/fnagi.2025.1639245)
Supplement: Supplementary file 1 [file Table_1.DOCX]

**Supplementary Table 1** Exercise-Cognitive Dual-Task Training Intervention Protocol

| **Serial Number** | **Posture** | **Physical Exercise Task** | **Cognitive Exercise Task** |
| --- | --- | --- | --- |
| 1 | Seated | Hamstring Stretch: Lean the body forward while extending one leg, taking deep breaths and focusing on the stretch in the hamstring of the extended leg. | Arithmetic Training  Count sequentially from 0, increasing by 2 each time (e.g., 0, 2, 4…).  Count backward starting from 90 (e.g., 90, 89, 88…). |
| 2 | Seated | Leg Lift and Knee Raise Exercise: Lift one leg while keeping the knee bent upwards, then slowly return to the starting position. Repeat the movement with the opposite leg. | Memory Training  Recall and enumerate:  The items in your refrigerator.  The names of provinces, cities, counties, or districts in your country.  What you had for dinner yesterday. |
| 3 | Seated | Toe and Heel Lift Exercise: Gradually lift and lower the toes, followed by lifting and lowering the heels in a controlled manner. | Attention Training  While scanning the room, answer the following:  How many circular objects are present?  How many red objects are visible?  How many green objects can you find? |
| 4 | Standing | Calf Muscle Stretch: Hold onto a table or chair for stability. Position both feet with toes pointing forward and gently stretch the calf muscles without applying excessive force. | Verbal Fluency Training  In 60 seconds, list as many words as possible from a single category:  Three-character phrases (e.g., Joyful Sheep, Bright Red).  Words related to summer (e.g., swimming).  Types of flowers (e.g., roses, carnations). |
| 5 | Seated | Chest and Back Stretch: Extend both arms outward and lift the chest, stretching the chest and back muscles. Take a deep breath in, then slowly relax while exhaling. | Memory Training  Recall and describe:  A recent news event.  Directions from your home to the nearest station.  The most delicious meal you have ever eaten. |
| 6 | Standing | Lunge Stretch: Use a table, chair, or handrail for support to maintain stability. Place one foot in front of the other while standing upright. Lower the body into a lunge position, ensuring the front leg forms a right angle with the thigh parallel to the ground. Keep the back heel elevated. Ensure the front knee remains above the toes and does not extend beyond them. | Arithmetic Training  Count sequentially starting from 2, increasing by 6 each time (e.g., 2, 8, 14…).  Divide even numbers by 2 repeatedly until division is no longer possible (e.g., 24 → 12 → 6 → 3).  Count backward starting from 300, gradually subtracting 2 or 3 once accustomed. |
| 7 | Standing | Lunge Stretch (Reiterated): Use a table, chair, or handrail for support to maintain stability. Place one foot in front of the other while standing upright. Lower the body into a lunge position, ensuring the front leg forms a right angle with the thigh parallel to the ground. Keep the back heel elevated. Ensure the front knee remains above the toes and does not extend beyond them. | Attention Training  While scanning the room, answer the following:  Are there any areas that require cleaning?  Are there any objects in the room that evoke nostalgia?  Are there scratches or marks on the walls? |
| 8 | Standing | Full-Foot Standing Balance Training: Lightly hold onto a sturdy table or chair for stability. Align the back foot’s toes with the heel of the front foot, standing as if on a straight line. Maintain this posture for 20 seconds, then switch the positions of the front and back feet and repeat the exercise. | Verbal Fluency Training  In 60 seconds, list as many words as possible from a single category:  Colors (e.g., red, blue).  Four-character idioms (e.g., easy-going, persistent effort).  Words related to winter (e.g., cold, snowflake). |
| 9 | Seated | Seated Coordination Training with Cognitive Function Exercise: Perform seated marching, clapping hands at multiples of 3 while counting. Follow the rhythm of counting by stomping feet and clapping hands at multiples of 3 (e.g., 3, 6, 9…) until reaching 30. | Advanced Exercises  : Follow the rhythm of your counting by stomping your feet and clapping your hands at multiples of 3 (e.g., 3, 6, 9…). Do not vocalize the number when clapping. Continue counting until reaching 30. |
| 10 | Standing | Standing Coordination Training with Cognitive Function Exercise: Perform stepping movements in rhythm with counting, clapping hands at multiples of 4 while counting. Follow the rhythm of counting by stepping and clapping hands at multiples of 4 (e.g., 4, 8, 12…) until reaching 40. | Advanced Exercises  : Follow the rhythm of your counting by stepping in place and clapping your hands at multiples of 4 (e.g., 4, 8, 12…). Do not vocalize the number when clapping. Continue counting until reaching 40. |
